# Presentation, Treatment, and Natural Course of Severe Symptoms of Urinary Tract Infections Measured by a Smartphone App: Observational and Feasibility Study

**DOI:** 10.2196/25364

**Published:** 2021-09-03

**Authors:** Akke Vellinga, Karen Farrell, Roisin Fallon, Daniel Hare, Una Sutton-Fitzpatrick, Martin Cormican

**Affiliations:** 1 School of Medicine National University of Ireland Galway Galway Ireland; 2 School of Medicine Health Research Board Primary Care Clinical Trials Network Ireland National University of Ireland Galway Galway Ireland; 3 Department of Clinical Microbiology University Hospital Galway Galway Ireland; 4 Tallaght University Hospital Dublin Ireland; 5 Health Service Executive Antimicrobial Resistance and Infection Control Team Dublin Ireland; 6 Discipline of Bacteriology National University of Ireland Galway Galway Ireland

**Keywords:** urinary tract infections, general practice, smartphone application, mobile phone

## Abstract

**Background:**

Urinary tract infections (UTIs) are one of the most common conditions in women. Current information on the presentation, management, and natural course of the infection is based on paper diaries filled out and subsequently posted by patients.

**Objective:**

The aim of this study is to explore the feasibility of a smartphone app to assess the natural course and management of UTIs.

**Methods:**

A smartphone app was developed to collect data from study participants presenting with symptoms of UTI in general practice. After initial demographic and treatment information, symptom severity was recorded by the patient after a reminder on their smartphone, which occurred twice daily for a period of 7 days or until symptom resolution.

**Results:**

A total of 181 women aged 18-76 years downloaded the smartphone app. The duration of symptoms was determined from the results of 178 participants. All patients submitted a urine sample, most patients were prescribed an antibiotic (163/181, 90.1%), and 38.7% (70/181) of the patients had a positive culture. Moderately bad or worse symptoms lasted a mean of 3.8 (SD 3.2; median 4) days, and 70.2% (125/178) of the patients indicated that they were cured on day 4 after consultation. This compares with other research assessing symptom duration and management of UTIs using paper diaries. Patients were very positive about the usability of the smartphone app and often found the reminders supportive. On the basis of the feedback and the analysis of the data, some suggestions for improvement were made.

**Conclusions:**

Smartphone diaries for symptom scores over the course of infections are an efficient and acceptable means of collecting data in research.

## Introduction

### Background

Urinary tract infections (UTIs) are one of the most common conditions for which women consult their general practitioner (GP) [1]. More than half of the women consult their GP for a UTI at least once in their lives [2,3].

UTIs are considered self-limiting infections [4,5], but antibiotics are generally prescribed empirically [6-8]. The prescription of antimicrobials for UTI accounts for a substantial amount of prescriptions in primary care and contributes to an increased risk of adverse drug effects, the burden of resistant infections [9,10], and increased costs [11]. In women presenting in primary care with suspected UTI, which turned out to be susceptible to the antibiotic prescribed, the duration of more severe symptoms was 3.3 days on average [2]. However, the duration increased to 4.7 days for women with resistant infections and 4.3 days for women with symptoms but no significant bacterial growth (urethral syndrome) [2]. An international comparison of the presentation and management of UTI in four European countries found wide differences in management, with antibiotics prescribed for 59%-95% of the presenting women; however, there were no differences in the time taken to resolve moderately bad to worse symptoms, which took a median of 4 days [12].

All studies reporting on the management of UTI base their findings on the use of paper diaries, which are provided to women at the time of consultation and are requested to be returned by post after symptoms are resolved [5,13-15]. Such diaries are also used in UTI trials where antibiotic treatment is compared with symptomatic treatment for UTI in general practice [16,17].

In one observational study that recorded the natural course of UTIs, 64% of the symptom diaries were recovered [2], and in another observational cohort of four countries, 70% of the diaries were recovered [18]. A Danish study observing the effect of point-of-care testing for UTI recovered 85% of the patient diaries [19]. All of these studies have used additional resources to contact patients and urge them to post their diaries.

Compliance with paper diaries has been compared with electronic diary keeping for patients recording pain three times daily for 21 days. The difference between electronic and paper recording was analyzed, and the results showed a high level of feigned compliance in the use of paper diaries [20]. Different versions of electronic diaries have been piloted and tested in various environments to record symptoms, such as in inflammatory bowel disease [21], overactive bladder [22], and asthma [23].

Smartphone apps are increasingly used in care innovation research and provide new opportunities to develop interventions [24]. In pain settings, apps have been most widely used to record diary entries both in observational [25] and clinical trial settings [26]; however, apps have also been implemented for other conditions [27-29]. In these studies, smartphone diaries have been shown to be highly efficient and generally perform better than paper-based diaries.

In our own SIMPle (Supporting the Improvement and Management of Prescribing for UTI) study, we used an observational study to follow patients with UTI using a smartphone app [30]. The results of this feasibility study showed that a smartphone app was well received and that once a patient committed to reporting their symptoms, they would continue doing so for the duration of the study [31].

### Objective

The objective of this study is to develop and evaluate the feasibility of a smartphone app to assess the natural course and management of UTIs.

## Methods

### Aim

This study explored the feasibility of a smartphone app to assess the natural course and management of UTIs in women presenting in primary care and to document the outcomes of urine culture, antibiotic resistance of bacterial growth, and antibiotic prescription using a smartphone app with reminders for data collection.

### Design

This study is an observational feasibility study of women presenting with symptoms of UTI.

### Setting and Participants

Between November 2018 and December 2019, 6 GPs in the west of Ireland enrolled adult female patients with symptoms of UTI. After obtaining consent, the patient received a patient number, which was texted to a central number to record the patient’s phone number, and a link to download the app was returned. Both patients and practitioners received a payment for each download of the smartphone app.

### Follow-up

A telephone call was made to all patients on days 4 and 28 (or as soon as possible after this day). The aim of the day 4 phone call was to confirm the symptom score. In addition, information on adverse events, experience using the app, and return consultation with a GP were recorded.

### Smartphone App

**Figure 1:**
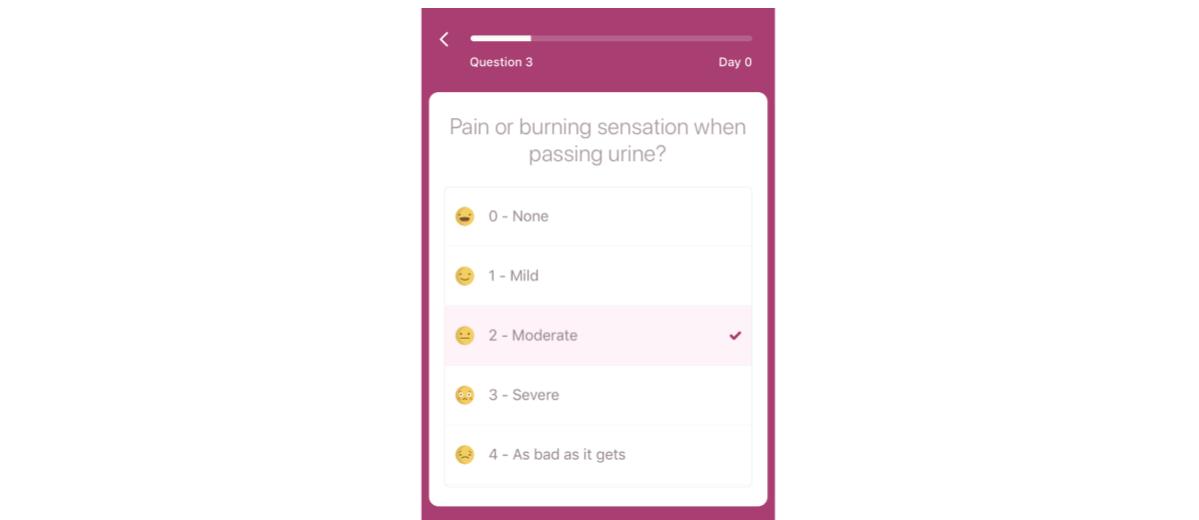
Smartphone app.

A smartphone app was developed to collect data from the participants (Figure 1). At enrollment, patients were requested to record demographic information (age, recent UTI, marital status, children, and medical card) as well as treatment details (antibiotic treatment: yes or no, name of antibiotic, and duration).

To record the severity of symptoms, patients received a reminder twice a day, once in the morning at 9 AM (or 10 AM and 11 AM if no entry was made) and once in the afternoon at 6 PM (or 7 PM and 8 PM if no entry was made). Patients were reminded to provide their symptom score for 7 days and, if no recovery occurred, up to 14 days or until symptom recovery. Symptom recovery was defined as a score of <2 (moderate) for each symptom.

Symptom severity included four symptoms: dysuria (painful and/or burning urination), frequency of urination, urgency of urination, and lower abdominal pain. Symptoms were scored from 0 (not at all) to 4 (as bad as it gets). An additional question—*Do you feel cured?*—was included after the symptom score to allow comparison with a Scandinavian trial in which this was the main outcome score [32].

On days 0 and 5, patients were also asked to fill out a UTI-related questionnaire on impairment to daily life activities on the Activity Impairment Assessment (AIA) [33] as well as self-rated health in general and on the particular day (on a visual analog scale from 0 to 100) [34]. The AIA is a five-item questionnaire that assesses the amount of times an individual’s work or regular activities has been affected because of their UTI. Patients respond to the AIA items on a five-point Likert scale, with the response options *none of the time, a little of the time, some of the time, most of the time,* and *all of the time*. The questions were as follows: did you have to cut down on time at work, could you accomplish less, were you limited in the kind of work you did, did you have difficulty performing work, and did your UTI interfere with social activity?

### Sample Size

Considering a mean difference of half a day clinically significant, a sample of 92 patients (α=.05 and β=.2) should detect such a difference in the mean duration of symptoms (score moderate or worse on the four items: dysuria, frequency of urination, the urgency of urination, and lower abdominal pain [5]) of 3.8 (SD 3.0) on day 4.

### Data Analysis

The duration of symptoms included the day the patient consulted their GP. The mean duration of symptoms was based on the day their mean total symptom score was moderately bad or worse and similarly for the duration of each symptom separately. The mean duration to cure is based on a yes answer to the question *Do you feel cured?* Symptom resolution was based on the day that all symptoms were 0. As the app was filled out for a duration of 7 days, the return of symptoms within the 7 days was included in the duration of symptoms. The end of symptoms was also indicated on the first day when a patient ceased making any more entries.

Antibiotic prescriptions were recorded on the day of the consultation, and patients were asked every day if they were still taking the antibiotic. The antibiotic prescribed was assessed retrospectively in the context of the microbiological culture results to assess if the organism tested was susceptible to the antibiotic chosen empirically.

Urethral syndrome was defined as having symptoms of suspected UTI, with no significant bacterial growth detected on culture [2].

Analysis was performed using STATA 13.0 (StataCorp LLC) and IBM SPSS, version 26. Tableau was used to visualize the data.

### Laboratory Analysis

Urine samples were collected from each patient. Samples were sent to the Microbiological Lab of the University Hospital Galway for analysis. A positive sample was based on a colony count of >10,000 cfu/ml pure growth observed after overnight incubation on chromogenic agar. Mixed growth was also recorded and included for further analysis. Organisms detected in pure culture were identified by matrix-assisted laser desorption ionization–time of flight (Bruker). Susceptibility testing was performed using the EUCAST (European Committee on Antimicrobial Susceptibility Testing) disk diffusion methodology and interpretive criteria.

### Ethical Approval

Ethical approval was obtained from the Irish College of General Practitioners. Individual informed consent was obtained from all participants before enrollment in the study. Consent had to be confirmed at registration when downloading the smartphone app.

### Availability of Data and Materials

Access to anonymized patient data can be obtained from the corresponding author upon request. The code for the smartphone app can be obtained from the corresponding author and used freely if referenced appropriately.

## Results

### Overview

A total of 181 patients downloaded the smartphone app. Their mean age was 29.7 (SD 14; median 22, range 18-76) years. Of the women, 28.1% (51/181) were married or in a relationship, 69.1% (125/181) did not have children, 18.2% (33/181) had one or two children, and 12.7% (23/181) had three or more children. More than one-third (67/181, 37%) of the patients were entitled to free medical and GP care, whereas 62.9% (114/181) had to pay for their GP visit as a private patient. Of the participants, 65.7% (119/181) had or were pursuing a university degree and 34.2% (62/181) had secondary education. A total of 39.8% (54/181) were working, 6.1% (11/181) were homemakers, and 54.1% (98/181) were students.

Of the 181 patients, 3 never filled out any symptom score, whereas all others provided at least one symptom score. On day 4, 39 did not provide a symptom score, but 13 of them provided one on the previous day.

Of the 181 patients, 163 (90%) were prescribed an antibiotic. Nitrofurantoin was most frequently prescribed (66.3%, 108/163), followed by trimethoprim (20/163, 12.3%), co-amoxicillin (11/163, 6.7%), amoxicillin (8/163, 4.9%), fosfomycin (5/163, 3.1%), and quinolone (2/163, 1.2%).

The question that was asked at every entry, that is, if they took their antibiotic or if they used any pain medication, was filled out irregularly and could not be analyzed.

### Symptom Duration

**Figure 2:**
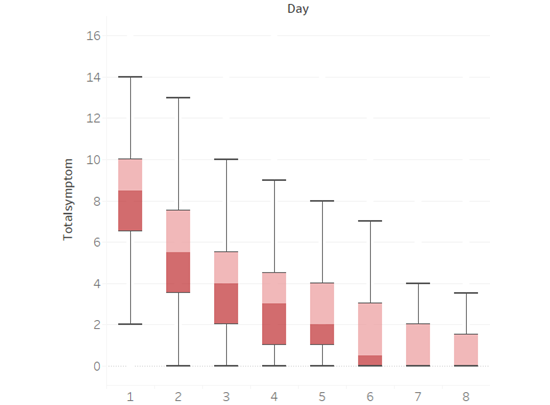
Total symptom score by day. Each box represents the interval of 50% of the symptom scores, with the difference in color at the mean score. Whiskers indicate the range of the symptom score on each day.

Symptoms started on average 5.6 days (SD 6.3) before they consulted their GP, with a median of 4 days (IQR 5). The mean duration of symptoms after they consulted their GP was 4.2 days (SD 3.0) and the mean time to cure (indicated by the patient) was 3.8 days (SD 3.2). Table 1 and Figure 2 show the mean duration of moderately bad or worse symptoms in more detail. The most common symptom rated moderately bad or worse on the first day was the urgency of urination (114/178, 64%), followed by frequency of urination (110/178, 61.8%), lower abdominal pain (98/178, 55.1%), and dysuria (71/178, 39.9%); the duration of each symptom is shown in Multimedia Appendix 1. There were no significant differences between the types of UTI and if an antibiotic was prescribed or not. An overview of the percentage of patients who indicated that they felt cured each day since the consultation is presented in supplementary Figure 2. Overall, 70.2% (125/178) of patients indicated that they felt cured on the fourth day after consultation (day 5), and this was 71.8% (115/160) among those who had an antibiotic prescribed.

**Table 1:** Mean (SD) and median of the duration of symptoms according to urinary tract infection type and antibiotic prescription (N=178).

Variables			Symptoms		Dysuria		Frequency of urination		Urgency of urination		Lower abdominal pain		Resolution		Cured
**Duration (days)**															
	Value, mean (SD)	4.2 (3.0)		2.0 (2.6)		2.6 (2.6)		3.0 (2.9)		2.9 (3.3)		10.7 (20.9)		3.8 (3.2)	
	Value, median	4		1		3		2		2		6		3	
**UTI** ^a^ **type,** **mean (SD)**															
	UTI sensitive to AB^b^ (n=36)	4.7 (3.5)		3.0 (0.6)		3.1 (3.3)		3.5 (3.5)		3.7 (3.6)		13.4 (26.3)		3.5 (3.1)	
	UTI resistant to AB or no AB (n=7)	5.0 (2.7)		3.6 (3.6)		3.4 (2.1)		3.4 (2.5)		3.0 (2.9)		6.3 (4.0)		4.3 (4.1)	
	UTI unknown sensitivity (n=26)	3.4 (1.8)		1.7 (1.6)		1.7 (1.2)		2.4 (1.7)		2.0 (1.9)		5.4 (2.2)		3.2 (2.0)	
	No significant bacterial growth (urethral syndrome; n=92)	4.3 (3.2)		1.6 (2.1)		2.8 (2.7)		2.9 (2.9)		3.1 (3.5)		12.0 (23.2)		4.1 (3.5)	
**Antibiotic, mean (SD)**															
	Yes (n=160)	4.3 (3.0)		2.0 (2.5)		2.7 (2.6)		3.0 (2.9)		3.0 (3.2)		10.3 (20.4)		3.8 (3.2)	
	No (n=18)	3.4 (3.5)		2.3 (3.6)		2.2 (2.6)		2.9 (3.5)		2.2 (3.6)		10.4 (22.3)		4.2 (3.6)	

^a^UTI: urinary tract infection.

^b^AB: antibiotic.

Overall, 90% (163/181) of patients had moderate to worse dysuria for 4 days or less, 85.1% (154/181) had a frequency of urination, 81.2% (147/181) had the urgency of urination, and 66.9% (121/181) had lower abdominal pain. Overall, symptoms lasted 4 days or less for 56.9% (103/181) of the patients after seeing their doctor (Multimedia Appendix 2).

### Microbiology

Overall, 38.7% (70/181) patients had a positive culture. Enterobacteriaceae (mainly *E. coli*) were cultured from 27.6% (50/181) of patients, followed by *S. saprophyticus* from 5.5% (10/181) of patients. A total of 17.7% (32/181) were mixed cultures and 12.2% (22/181) were <10,000 cfu/ml. Resistance to at least one of the tested antibiotics was recorded in 61% (33/54) of isolates for which this could be assessed. Comparing the antibiotic prescribed with the isolate and its resistance showed that 55% (36/66) of the isolates tested were susceptible to the antimicrobial prescribed, whereas for 41% (27/66), this was not known. For 3 patients, the cultured organisms tested resistant to the antibiotic prescribed. One of these patients was contacted to change the antibiotic (from co-amoxyclav to nitrofurantoin).

### AIA and General Health

**Figure 3:**
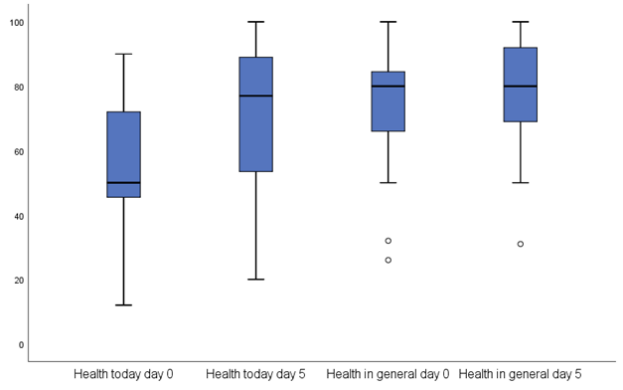
Health score indicated on a sliding scale (0-100) answered for the questions how is your health today? and how is your health in general on day 0 and day 5. The box represents 50% of the answers, whereas the whiskers cover 100% of the answers. The line in the box represents the mean score. Outliers are indicated by the dots.

Little difference was observed for the AIA score (Table 2); the mean AIA score on day 0 was 6.6 (SD 5.0), and on day 5, it dropped to 4.1 (SD 4.6). A paired two-sided *t* test of patients providing a score on both days showed significance (*P=*.03), and patients were asked to rate their general health on day 0 (mean 75.6, SD 17.3) and day 5 (mean 76.3, SD 18.2; Figure 3). Their health was rated at 55.8 (SD 20.1) on day 0 and 71.9 (SD 19.0) on day 5. The difference between their rated health on days 0 and 5, as well as the difference in overall health, was significant. On day 5, their health scores were still significantly different from the overall health scores, but the overall health scores on days 0 and 5 were not statistically different.

The total symptom score on day 0 was correlated with the AIA (R^2^=23%) and similarly with the health score (17%) on day 0 and day 5.

**Table 2:** Overview of the Activity Impairment Assessment (urinary tract infection–related questionnaire on impairment in daily life activities on the Activity Impairment Assessment) and self-rated health in general and on the particular day (on a visual analog scale from 0 to 100).

		Day of consultation (n=111), mean (SD)	Day 5 (n=96), mean (SD)
**AIA^a^ item**			
	Cut down on time at work	0.95 (1.2)	0.6 (1.0)
	Accomplished less	1.5 (1.2)	0.8 (1.0)
	Limited in type of work	1.2 (1.2)	0.7 (1.0)
	Difficulty performing work	1.4 (1.1)	0.8 (1.0)
	Interfered with social activity	1.6 (1.2)	1.1 (1.2)
Total AIA score		6.6 (4.9)	4.1 (4.6)
Health score in general		76 (17)	76 (18)
Health score today		56 (20)	72 (19)

^a^AIA: Activity Impairment Assessment.

### Associations

There was no difference in duration of symptoms, total symptom score on the day of consultation, AIA, and health status on the day of consultation between those who did and did not go to university or between students and nonstudents. Only the total symptom score was significantly higher (*P*=.03) for those not in a relationship compared with participants who were married or with a partner, but no other differences were observed. Age was not correlated with any outcome (duration, total score, days with symptoms before consultation, health score, or AIA).

### Day 4 and Day 28 Phone Call

A call was answered by 152 participants on day 4, and 128 patients answered on day 28.

On day 4, 39.8% (71/178) indicated that they felt cured, compared with 70.2% according to the app data. A comparison showed that 67.7% (103/152) indicated the same on the call as they did on the app, 3.9% (6/152) indicated that they were cured over the phone but not on the app, and 41/152 (26.9%) said they were cured in the app but not when asked over the phone. The total symptom score on day 4 according to the phone call was 3.8 days (SD 3.5) according to the call and 2.6 days (SD 2.6) according to the app, which was significantly different, that is, symptoms were rated more severe when asked over the phone.

When asked about side effects, 28.9% (44/152) reported side effects from the medication. The main side effects recorded were stomach upset or diarrhea (17/152, 11.2%) and thrush (4/152, 2.6%), and 18 did not specify any side effects, and five reported urine discoloration (all patients reported that they had received nitrofurantoin).

Of the patients who answered a call on day 28, 25% (32/128) consulted the GP again for symptoms of UTI during the 28 days after the initial consultation. Patients who consulted their GP had significantly (*P*=.006) higher symptom duration (5.7 days, SD 4.3 vs 3.9 days, SD 2.6) but no difference in symptom score on the day they consulted. There was no difference in antibiotic or painkiller prescription between those who did and did not reconsult. Patients who reconsulted were significantly older (mean 35.9, SD 17.8 vs mean 27.9, SD 12.4). On day 5, the AIA score (6.9% vs 2.9%) was significantly higher, and the health score (59.7% vs 75.1%) was significantly lower for those who reconsulted.

### Evaluation of the App

On the day 4 call, 116 patients provided further comments on their UTI and/or the use of the app, whereas 128 patients provided further comments on their UTI and/or the use of the app on day 28. In general, the comments were positive. Some patients liked the fact that the app reminded them of their medication and improvement of their symptoms:

Handy for reminding in morning and evening to take my medication and think about my symptoms more and if they were improving or not.

App was good as made me check in with myself, showed me how it was a recovery process.

App was good, not time consuming, interesting for herself to track day to day feelings.

Overall, the app was received well:

App was good, quick and easy to use.

However, a few comments were made in relation to the repetition of questions:

Felt like they were the same questions over and over again.

## Discussion

### Principal Findings

It was shown that the use of a smartphone app to track patients’ symptoms of UTI is an efficient and effective approach to replace paper diaries. Our estimates of the mean and median duration of symptoms from the date of the consultation were 4.2 days and 4 days, respectively. This is comparable with the observations made in previous studies of the natural course of infection in which paper diaries were used [2,12]. However, the mean and median duration of individual symptoms were rated lower in the app than in the paper diaries from both studies. Comparing our results with other trials using paper diaries showed a mean duration of moderate to worse symptoms at 4.6 days [16] compared with 4.3 in our study. The *do you feel cured* question as the main outcome in the Scandinavian trial was also included in our study; 74% indicated cured on day 4 after the consultation when taking the antibiotic, whereas in our study, 71.8% indicated cured in the group where an antibiotic was prescribed. In general, the outcomes are similar to the outcomes of other studies. The main difference and challenge in comparing different studies and trials is the day on which outcomes are measured, that is, whether this includes the day of consultation or not. Inclusion of the method of measuring in a core outcome set for UTI should be clearly defined [35].

UTIs are considered self-limiting infections [4,5], but antibiotics are generally prescribed empirically [6-8]. The prescription of antimicrobials for UTI accounts for a substantial number of prescriptions in primary care, contributing to an increased burden of resistant infections [9,10] as well as increased costs [11]. Our study showed that 90% of the patients presenting were prescribed an antibiotic, of whom 81.6% received a first-line antibiotic [8]. Only 38.7% of the patients had bacteriological confirmation of UTI. Very similar figures of prescribing were confirmed in Wales, England, and Spain, whereas the Netherlands showed much lower prescribing (59%); however, confirmed UTI was approximately 17% in Wales and England, 42% in Spain, and 64% in the Netherlands. Although more literature is emerging suggesting that symptomatic treatment of UTI is often sufficient and acceptable [16,17], most GPs are hesitant to do so. Compared with the last 10 years, GPs are prescribing more in line with guidelines. In 2010, in a study conducted in the same region, findings suggested only 26% GPs prescribing a first-line antibiotic [7]. The efforts made since then to decrease the use of co-amoxyclav in general practice and encourage the use of (first line) prescribing guidelines seem to be having an effect [30,36].

Our overall AIA score on the day of the consultation was 6.6 and 4.1 on day 4, compared with the trial of Gagyor et al [16], in which 8.9 and 1.1 were recorded, respectively. However, it is unclear why such large differences were observed. The validation of the AIA score showed a total score of 7.6 on the day of consultation, which is still higher than our finding, but they do not provide a follow-up score [33]. The inclusion of the health score might better measure general well-being on the day of the consultation, and it shows high concordance between the general health score on day 0 and day 4. This general health score may provide a better insight into the severity of the UTI and its impact on well-being.

The day 4 phone call was included to see how the smartphone scores compared with the answer provided over the phone, and 39.8% indicated that they felt cured, compared with 70.2% indicating to be cured when recorded on their smartphone. The total symptom score according to the app was also lower than that according to the phone call. Again, it is not clear why people report lower scores on the app than on a phone call.

During the follow-up period, 25% reported a reconsultation and 28.9% reported side effects of some sort, which is in line with the reported reconsultations in the four-country study.

In relation to the use of a smartphone app to collect data and considering the comments as well as the analysis of the data, a number of conclusions can be made (Textbox 1).

**Conclusion made based on the use of a smartphone app to collect data, comments, and analysis of the data.**

**Conclusions**

- A smartphone app is an efficient and effective tool for collecting real-time data in research on infections.
- Our app asked for daily scores twice, which were decided to capture differences between morning and afternoon scores. Many did not fill out the app twice a day, and those who did, did not show major differences between morning and afternoon. It also introduced an additional step in the analysis, using the mean score for the day, or if only one score was provided, to use this score.
- Patients were interested and committed to using their app at the start of the study. At this stage, a few additional questions could be included to improve its use:
- The app should request at the start what time of the day is most convenient to fill out the app. This should then be the trigger for the reminder on the phone to fill out their app.
- Type and duration of antibiotic prescribed: On the day after their course is intended to finish, patients can be asked if they have any medication left. This would avoid repeatedly asking if they had taken their antibiotic.
- For painkiller use, this should only be included if it is part of particular interest to the main research question.

### Strengths and Limitations

To our knowledge, this is the first prospective study to describe the natural course of a UTI using a smartphone app. Smartphone apps are frequently used for chronic conditions, where patients have more time and are not in distress when asked to enroll in a study. The acceptability and ease of use of the smartphone app have been shown for both patient use and completeness of data. However, participants may have been selectively rather than randomly invited to participate, but no reliable logs of potentially eligible patients were retained. We encouraged GPs to enroll any age group, and the age range (18-76 years), clearly shows that age was not a limitation to participate in our study. The overall mean age of participants was, however, younger than previous studies by our group, in which the mean age was 56.1 years (SD 20.7), and is probably due to the inclusion of a student GP center for enrolment of patients [30]. In the pilot study of our smartphone app to record symptoms of UTI, we found GPs to favor inviting younger patients to participate, which we subsequently and in this study, aimed to avoid by asking GPs to include all ages [31]. The restrictions to enrollment are most likely because of the additional time necessary to consent patients to the study, particularly as UTI consultation is usually considered straightforward and short.

A few questions arise with the results obtained, particularly in relation to discrepancies between phone call scores and smartphone scores from the same day. Other research into the course of infections using a smartphone app may be performed in the future, which may shed more light on this. However, no other study has identified the use of smartphones for infection symptom scores, so our findings could not be confirmed. However, the similarities between our outcomes and those of studies with paper diaries are encouraging. In particular, for trials where consent will be required anyway, smartphone diaries may be considered in the future, which could significantly reduce the energy and time spent to collect outcomes after a patient leaves the practice.

### Conclusions

Smartphone diaries for symptom scores over the course of infections are an efficient and acceptable means of collecting data in research.

## Appendix

Multimedia Appendix 1 Mean duration of each symptom and total symptom scored moderate to severe for each symptom (dysuria, frequency of urination, urgency of urination, and lower abdominal pain), and duration for any of these symptoms (average total duration).

Multimedia Appendix 2 The day a patient indicates to be cured from the day of consultation.

## Abbreviations

AIA: Activity Impairment Assessment
EUCAST: European Committee on Antimicrobial Susceptibility Testing
GP: general practitioner
SATIN: Symptomatic Versus Antibiotics Treatment of Urinary Tract Infections in Women
SIMPle: Supporting the Improvement and Management of Prescribing for Urinary Tract Infection
UTI: urinary tract infection
