# Supplementary figures and images for: Presentation, Treatment, and Natural Course of Severe Symptoms of Urinary Tract Infections Measured by a Smartphone App: Observational and Feasibility Study
Source: J Med Internet Res. 2021 Sep 3;23(9):e25364. doi: 10.2196/25364 (PMC8449294; doi:10.2196/25364)

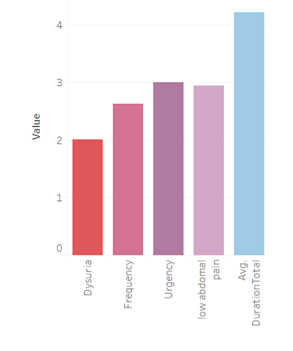

Supplement: Multimedia Appendix 1 [file jmir_v23i9e25364_app1.png]

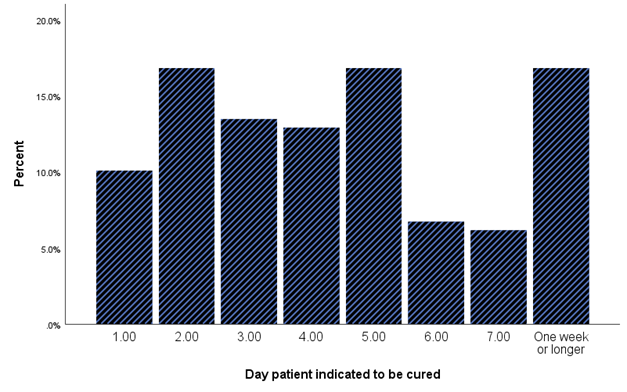

Supplement: Multimedia Appendix 2 [file jmir_v23i9e25364_app2.png]
